# Neonatal pain in a broader perspective: an analysis through systems thinking

**DOI:** 10.1590/1980-220X-REEUSP-2025-0399en

**Published:** 2026-03-20

**Authors:** Silvia Naujorks, Julieli Rosso, Luana Pizarro Meneghello, Dirce Stein Backs, Regina Gema Santini Costenaro

**Affiliations:** 1 Universidade Franciscana, Santa Maria, RS, Brazil.

**Keywords:** Pain, Infant, Newborn, Intensive Care Units, Neonatal, Health Promotor

## Abstract

This essay aims to reflect on the complexity of neonatal pain in light of Fritjof Capra’s systemic thinking. It analyzes the phenomenon of pain in newborns admitted to neonatal intensive care, highlighting its complex and multidimensional nature. It observes the main characteristics of the neonatal system that guide and support multidisciplinary care from a systemic perspective. A parallel is drawn between the reductionist view, characterized by punctual and isolated interventions, and the view of pain from the perspective of complexity, which leads to integrality, multidisciplinarity, and interconnectivity. Systemic-complex thinking is established as a relevant framework for understanding the multidimensionality of pain perception by newborns admitted to neonatal intensive care units and by care teams, aiming at integrated, multidisciplinary, humanized care sensitive to the particularities of the newborn and their environment. The implications for neonatal nursing include the need for continuous and contextualized pain assessment, the development of competencies for integrated management, and leadership in coordinating multidisciplinary strategies.

## INTRODUCTION

The International Association for the Study of Pain (IASP), in 2020, defined pain as an unpleasant sensory and emotional experience, not necessarily associated with actual tissue damage. It is a complex experience, resulting from the interaction of the immune, nervous, and endocrine systems, and can be influenced by previous painful experiences^(1)^.

Newborns (NBs) admitted to Neonatal Intensive Care Units (NICUs) are a high-risk population due to the fact that they are subjected daily to potentially painful procedures during their hospitalizations. In addition, their nonverbal nature and immaturity make it difficult to correctly identify and manage these painful stimuli, resulting in high rates of potentially painful procedures performed without any type of analgesia^(2)^.

Many studies have demonstrated short- and long-term consequences that repeated and untreated painful stimuli can result in, ranging from low growth rates to alterations in brain, cognitive, motor, and visual development, leading to poorer outcomes in school ages^(3,4)^. In addition to these, there are the immediate effects of painful stimuli, which include tachycardia, increased blood pressure, altered coagulation factors, and oxidative stress, which together can increase the risk of intracranial hemorrhage and mortality^(1)^.

Thus, given the vulnerability of this population, the healthcare team providing care to the newborn (NB) must have a broad and extensive vision, in order to understand the uniqueness and multidimensionality of pain therapy, through their qualification and grounded in systemic references^(5,6)^.

Systemic thinking considers interprofessional approaches, based on a unique and multidimensional understanding. Only with a broad approach – which considers the interdependence and intertwining of all phenomena in the neonatal system, which is multidisciplinary and with collaborative management strategies – will it be possible to understand the phenomenon of neonatal pain^(6,7,8)^.

In light of the above, this essay aims to develop a theoretical reflection on the complexity of pain through the lens of Fritjof Capra’s systemic thinking. Capra understands complexity as a departure from Cartesian and reductionist perspectives toward a systemic worldview, in which reality is conceived as an integrated whole formed by a dynamic web of interrelated events.

## METHOD

This theoretical-reflective study analyzes the phenomenon of pain in newborns admitted to the NICU, from a systemic perspective, with the aim of providing a basis for the construction of care that promotes their unique and multidimensional comfort and well-being. Systemic-complex thinking is established as a relevant reference for understanding the multidimensionality of pain perception by newborns admitted to the NICU and by the care teams, aiming at integrated, multidisciplinary, humanized care that is sensitive to the particularities of the newborn and their environment.

Fritjof Capra, a physicist and thinker known for promoting systemic thinking, emphasizes the importance of understanding living beings as complex, contextual, and unique, forming part of a web of relationships, in which all phenomena are intertwined and interdependent. He emphasizes that, in order to understand pain and be able to alleviate it, we must consider it in its broader context, which includes not only its physical basis, but also its emotional and environmental basis, as well as its interconnections. Thus, the present study is outlined based on the theoretical- reflective framework of systemic thinking from the productions of Fritjof Capra^(9,10)^ and systemic reference authors cited by him, in order to ground a more humanized, multidimensional, singular and interactive neonatal care approach, which is carried out through an integrated multiprofessional action.

## NEONATAL PAIN: A COMPLEX EXPERIENCE

The study of neonatal pain is relatively recent. It was demonstrated, only after 1980, that newborns are capable of feeling pain, including extremely premature infants, breaking with the paradigm that because they have immature organs and connections they would not be able to experience this. Since then, many studies have been published showing the consequences resulting from prolonged and unmanaged exposure to painful stimuli, as well as ways to better identify and manage it^(1,2,11)^.

In the study of pain, the very nature of the nervous system with its intricate network of neurons and communication pathways corroborates the need for a systemic view of the subject. Its multiple connections and feedback mechanisms make the painful experience unique for each human being. Given its complexity, pain cannot be restricted only to a physical cellular phenomenon, but rather to a network of interactions between the biological, emotional, psychological and social spheres^(2)^. Thus, its study transcends the scope of a reductionist structure, requiring a view that considers the totality of the individual and their interactions with the environment^(12,13)^. The mechanisms involved in the phenomenon of pain, or nociception, are considered adaptive responses of the body to something potentially harmful. These responses occur after stimulation of nociceptive neurons sensitive to thermal, mechanical, or chemical stimuli, which contain and release neuropeptides and are influenced by hormones and the immune system.

For a nociceptive stimulus to be perceived, four processes are required: transduction, in which peripheral nociceptors convert noxious stimuli into action potentials; transmission, whereby these signals travel through peripheral nerves to the spinal cord; perception, during which the stimulus ascends via the spinothalamic tract to the cerebral cortex, where it is consciously experienced as pain; and modulation, involving descending pathways that regulate the intensity of the ascending signal, notably through the action of endogenous opioids^(14)^. Together, these processes enable the nociceptive system to play a crucial role in survival by allowing the body to detect, respond to, and interact with potentially harmful environmental stimuli^(15)^.

Painful or stressful stimuli induce a physiological response, which includes an increase in circulating catecholamines, blood pressure, intracranial pressure, and heart rate, demonstrating the interconnectedness of all systems in pain perception. In premature infants, this response is less competent, which makes indications of behavioral changes and vital signs unreliable in recognizing pain. Added to this is the fact that persistent painful stimuli exhaust the response of the sympathetic nervous system, obscuring the signs of pain or discomfort^(1,2,16)^.

The impact of unmanaged neonatal pain reaffirms its systemic nature, as it causes everything from changes in local sensitivity and low growth rates to structural brain changes, such as: reduction of cerebellar and thalamic white matter, with consequent worse cognitive, motor and visual outcomes. Added to these situations are worse clinical outcomes, with a consequent increase in morbidity and mortality. Therefore, a systemic approach to the subject becomes imperative, as an important measure to reduce neonatal pain and infant mortality^(17,18,19)^.

There are many stimuli that can be perceived as painful by neonates, ranging from invasive procedures (punctures, catheterizations, probes), associated pathologies (enterocolitis, meconium aspiration syndrome, pneumonias, infections), to environmental factors (excessive light, noise), in addition to the act of care itself performed by the care team. Extremely premature infants (born before 28 weeks of gestational age) are an even higher risk population for repeated painful procedures, due to their length of stay in the NICU and their pathologies associated with prematurity. Thus, it is necessary to use a care improvement strategy capable of taking into account all these aspects of the painful stimulus^(1)^.

The nonverbal nature of newborns has led to the development of assessment scales designed to aid in the identification of pain by evaluating physiological and behavioral changes associated with noxious stimuli. These tools constitute only one component of the assessment process and must be applied within the context of the individual newborn, as more immature infants may exhibit diminished or absent responses due to neurological immaturity or chronic exposure to pain. Moreover, the quality of pain assessment and management is also influenced by the level of knowledge and training of the healthcare team^(2,4)^.

The National Policy for Humanized Care of Newborns – Kangaroo Method, established by the Ministry of Health, advocates a perinatal care model focused on quality and humanization of care, as well as welcoming. This policy recognizes that neonatal care should go beyond biomedical aspects, incorporating emotional, family, and environmental dimensions. Regarding the management of neonatal pain, the policy emphasizes the need for integrated strategies that include non-pharmacological measures, such as skin-to-skin contact, breastfeeding, non-nutritive sucking, and the reduction of harmful stimuli, in addition to pharmacological interventions when necessary.

The document also highlights the importance of ongoing training for healthcare teams for the early identification and appropriate management of pain, considering the particularities of each newborn and promoting an environment conducive to neuropsychomotor development^(20)^. This approach is aligned with the principles of neuroprotection, which aim to minimize brain injuries and optimize the development of the central nervous system during the hospitalization period^(21)^. Despite this, currently validated pain assessment scales do not incorporate the environment of each newborn into their metrics, which can lead to errors in the identification and management of pain phenomena.

Therefore, we need a multidimensional instrument constructed from the perspective of complexity thinking, that is, flexible, allowing for real-time adaptations and considering physiological, emotional, and environmental factors, to be used as a guide by the care team. The implementation of management strategies should be multidisciplinary, promoting a collaborative approach that recognizes the interdependence of the factors involved in the painful experience^(6,7,8)^.

**Figure 1:**
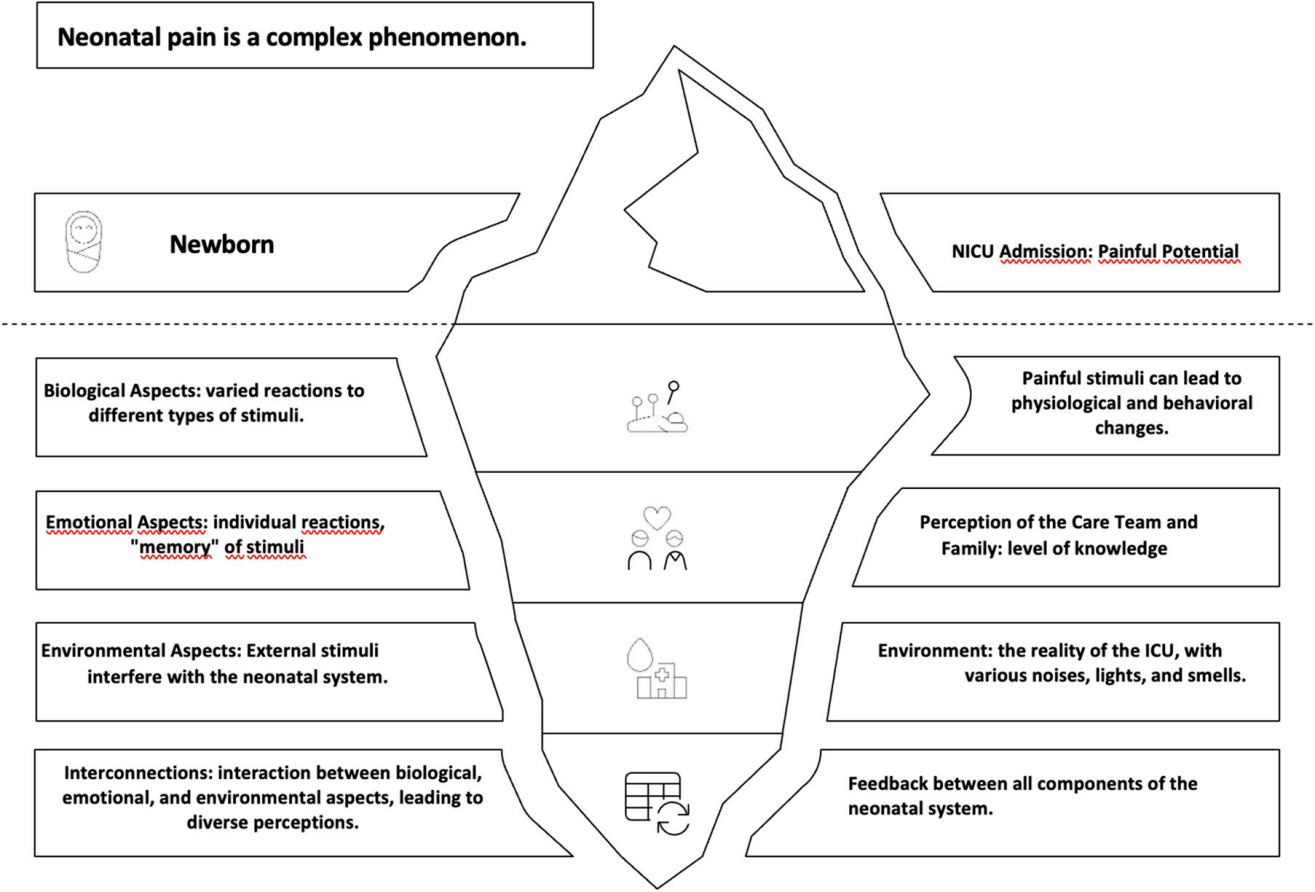
Characteristics of neonatal pain.

Source: Prepared by the author.

The adoption of a complex perspective in the assessment of neonatal pain demands a paradigm shift in clinical practice, with the training of a team capable of understanding and acting on the network of interactions that make up the neonatal system (Figure 1).

## NEONATAL PAIN FROM A SYSTEMS THINKING PERSPECTIVE

In the thinking of the physicist and systems theorist, Fritjof Capra, a living phenomena can only be understood through a paradigm shift, deconstructing the mechanistic, Cartesian and reductionist view of Descartes and Newton and reconstructing a systemic worldview. In this new view or approach, the world is seen as an integrated whole and not as a collection of dissociated parts, through the fundamental interdependence of all phenomena^(9)^.

The systemic view of life, called the new paradigm, deals with a holistic view of the world, or rather, a deep ecological view, where living systems, in addition to being seen as an integrated whole and not just a collection of parts, are embedded in their natural and social environment, physically and spiritually, in a dynamic balance. There is an intertwining and interdependence of all phenomena, through the conception of living systems as composed of networks, where there are no hierarchies, only networks nested within other networks. The universe is seen as a dynamic web of interrelated events, where all scientific conceptions and theories are limited and approximate^(10)^.

Regarding the human nervous system, Capra describes it as dynamic and continuously changing, through the interaction of processed information with the environment. Thus, human intelligence, memory, and decisions are never completely rational, but colored by emotions, with all knowledge derived from experience, that is, unique to each individual. The circular organization of the nervous system and its self-organizing and self-referential characteristic leads to the understanding that perceptions cannot be seen as mere representations of an external reality, but the continuous creation of new relationships within the neural network^(22,23)^.

With regard to cognition, that is, the process of knowing, we have mental activity as the organizing activity of living systems at all levels. It manifests itself not only in individual organisms, but also in social systems and ecosystems. Thus, Santiago’s theory, developed by Maturana and Varela, states that a brain is not necessary for the mind to exist, broadening the concept of thinking and encompassing the entire process involving perception, emotion, and action. In this premise, even the simplest organisms are capable of cognition, reacting to the environment in which they are^(9)^.

The role of stress and emotional states in the course of diseases is now well documented, with the care team responsible for dealing with the patient as a whole and with their relationship with the physical and emotional environment. The phenomenon of pain itself is still poorly understood due to the limitations of scientists and health professionals in integrating physical and psychological elements. Research still presents gaps related to the precise factors causing pain, which is why the communication pathways between the body and the mind are not yet well understood. In practice, it is almost always impossible to distinguish which are the sources of biological pain and which are the sources of psychological pain in two patients with the same physical symptoms, one may be suffering excruciating pain while the other feels little^(24)^.

Thus, to better understand and, consequently, better manage the phenomenon of pain, it must be considered in its broader context, which includes attitudes, mental expectations of the patient, their belief system, family support, and many other circumstances. Current medical practice tends to limit pain to some specific physiological disorder, where it is often denied or suppressed with analgesics alone. Therefore, stress reduction, in addition to pain processes, should be part of the treatment, aiming at the healing process^(9)^.

The persistence of a patriarchal view of power in the health care system, centered on the figure of the doctor, often prevents the rest of the team from using their full potential for the healing process. The important role that nurses play, for example, in the healing process, through close contact with patients, is not fully recognized. Hence the importance of a multidisciplinary and comprehensive approach, which goes beyond scientific assessment, but considers the broader knowledge of the physical and psychological state, through an integrated view of the patient’s emotional state, family history and social situation^(6,7,8,9,25)^. In systems science, living systems cannot be understood through analysis, as the properties of the parts are not intrinsic properties and can only be understood within the context of the larger whole. Thus, systems thinking is contextual and environmentalist, taking into account the environment in which a given system is embedded. In it, multiple interconnections, feedbacks, and non-linear processes occur, producing emergent behaviors that are impossible to predict through fragmented analysis^(10)^. Following this approach, pain should be studied and evaluated as an integrated phenomenon shaped by the external environment. A comprehensive understanding therefore requires an approach that acknowledges its complexity and the interconnection of physiological, neurological, environmental, and emotional factors (Chart 1).

**Chart 1:** Parallel between reductionist and complexity views – Santa Maria, RS, Brazil, 2025.

Aspect	Reductionist view	A view of complexity
**Overview**	He views pain as a simple, linear, and isolated phenomenon.	Views pain as a complex, multifaceted, and interconnected phenomenon.
**Theoretical basis**	Influenced by Newton’s classical physics, which views the world as a direct cause and effect.	Inspired by systems theory, which values the interactions and interdependence of elements.
**Treatment approach**	It focuses on specific, mechanical interventions.	It adopts a multidisciplinary approach, considering physiological, emotional, social, and environmental factors.
**Acknowledging the pain**	It underestimates or neglects the subjective experience of the newborn.	It recognizes pain as a complex experience, influenced by various contextual factors.
**Newborn care**	It treats the newborn as a mechanical system, without considering its environment or emotions.	Views the newborn as a whole being, with emotional, social, and environmental needs.
**Clinical practice**	Isolated interventions, often without considering the full context.	Integrated, humanized interventions that are sensitive to the specific needs of newborns and their environment.
**Example of a strategy**	Use of specific medications to relieve pain.	Use of multidisciplinary strategies, including environmental, emotional, and physiological care.

Source: Prepared by the author.

Applying these concepts to the neonatal ecosystem, one can understand why neonatal pain cannot be reduced to isolated alterations in physiological signs or to a single cause. It must be perceived as the product of an intricate network of interactions between the newborn’s nervous system, the hospital environment in which they are located, the actions of the care team, and the emotional context of the newborn and their family. Therefore, excessive or inappropriate sensory stimuli, such as bright lights and noises, can lead to alterations in the newborn’s physiological state, which in turn can influence their behavioral response and their subjective experience of pain. Furthermore, feedback between these elements can also create a dynamic cycle where small changes can generate amplified or attenuated effects over time. Understanding the neonatal system as a whole promotes actions that favor the stability, comfort, and well-being of the newborn, taking into account its interconnections.

## CONCLUSION

Due to its complex nature, pain cannot be effectively identified through the isolated application of assessment scales without consideration of other components of the neonatal ecosystem, many of which are not captured by the metrics currently validated and used in neonatal intensive care units.

Neonatal nursing occupies a strategic position in the identification, assessment, and management of pain, due to its continuous proximity to the newborn and their family. To this end, competencies must be developed for a contextualized assessment of pain, which transcends the mechanical application of scales and incorporates the careful observation of the environment, subtle behavioral responses, and clinical trajectory, which requires continuing education and clinical competence to recognize atypical signs, especially in extremely premature and critically ill neonates. By also leading the implementation of non-pharmacological strategies and the articulation and coordination of multidisciplinary actions, it assumes a leading role in reducing neonatal suffering and promoting better short- and long-term outcomes.

Adopting a systems-thinking approach to neonatal pain requires a reexamination of assessment and management strategies. Moreover, it encourages the development of collaborative care teams that acknowledge the relevance of each element within the system to the newborn’s pain experience and promotes research that embraces the phenomenon’s complexity by integrating physical, social, and emotional dimensions of pain perception. In this context, Fritjof Capra offers a robust theoretical foundation by emphasizing systemic interconnection, dynamism, and emergence, framing the newborn as a complete system in continuous interaction with their environment.

## DATA AVAILABILITY

The entire dataset supporting the results of this study was published in the article itself.
