# Supplementary material for: Neonatal pain in a broader perspective: an analysis through systems thinking
Source: Rev Esc Enferm USP. 2026 Mar 20;60:e20250399. doi: 10.1590/1980-220X-REEUSP-2025-0399en (PMC13004110; doi:10.1590/1980-220X-REEUSP-2025-0399en)
Supplement: Translation

# Translation: A dor neonatal em perspectiva ampliada: análise à luz do pensamento sistêmico

**DOI:** 10.1590/1980-220X-REEUSP-2025-0399pt

**Authors:** Silvia Naujorks, Julieli Rosso, Luana Pizarro Meneghello, Dirce Stein Backs, Regina Gema Santini Costenaro

**Affiliations:** 1 Universidade Franciscana, Santa Maria, RS, Brasil.

**Keywords:** Dor, Recém-Nascido, Unidade de Terapia Intensiva Neonatal, Promoção da Saúde

## Abstract

Objetiva-se refletir sobre a complexidade da dor neonatal à luz do pensamento sistêmico de Fritjof Capra. Ensaio teórico que analisa o fenômeno da dor em recém-nascidos internados em terapia intensiva neonatal, destacando sua característica complexa e multidimensional. Observa-se as principais características do sistema neonatal que conduzem e sustentam o cuidado multiprofissional na perspectiva sistêmica. Concebe-se um paralelo entre a visão reducionista, esboçada por intervenções pontuais e isoladas, e a visão da dor na perspectiva da complexidade, que conduz à integralidade, à multidisciplinaridade e à interconectividade. O pensamento sistêmico-complexo estabelece-se como um referencial relevante à compreensão da multidimensionalidade da percepção dolorosa pelos recém-nascidos internados em unidade de terapia intensiva neonatal e pelas equipes assistenciais, visando ao cuidado integrado, multidisciplinar, humanizado e sensível às particularidades do recém-nascido e seu ambiente. As implicações para a enfermagem neonatal incluem a necessidade de avaliação contínua e contextualizada da dor, o desenvolvimento de competências para manejo integrado e a liderança na coordenação de estratégias multidisciplinares.

## INTRODUÇÃO

A Associação Internacional de Estudos da Dor (IASP), em 2020, definiu a dor como uma experiência sensitiva e emocional desagradável, não sendo necessariamente associada a uma lesão tissular real. É uma experiência complexa, que resulta da interação dos sistemas imunológico, nervoso e endócrino, e que pode ser influenciada por experiências dolorosas anteriores^(1)^.

Os recém-nascidos (RNs) internados em Unidade de Terapia Intensiva Neonatal (UTIN) são uma população de risco devido ao fato de serem submetidos diariamente a procedimentos potencialmente dolorosos durante suas internações. Além disso, a sua natureza não verbal e imaturidade dificultam a correta identificação e o manejo desses estímulos álgicos, o que resulta em altos índices de procedimentos potencialmente dolorosos realizados sem qualquer tipo de analgesia^(2)^.

São muitos os estudos demonstrando consequências a curto e longo prazo que os estímulos álgicos repetidos e não tratados podem resultar, desde baixas taxas de crescimento até alterações de desenvolvimento cerebral, cognitivo, motor e visual, levando a piores resultados em idades escolares^(3,4)^. Soma-se a esses, os efeitos imediatos dos estímulos dolorosos, que incluem taquicardia, aumento pressórico, alteração de fatores de coagulação e estresse oxidativo que, em conjunto, podem aumentar o risco de hemorragia intracraniana e mortalidade^(1)^.

Assim, em vista da vulnerabilidade dessa população, cabe à equipe de profissionais de saúde, que presta assistência ao recém-nascido (RN), uma visão ampliada e colegiada, de modo a apreender a singularidade e multidimensionalidade terapêutica da dor, através da sua qualificação e embasamento em referenciais sistêmicos^(5,6)^.

O pensamento sistêmico considera abordagens interprofissionais, a partir de uma apreensão singular e multidimensional. Somente com uma abordagem ampla – que considere a interdependência e o entrelaçamento de todos os fenômenos no sistema neonatal, que seja multidisciplinar e com estratégias de manejo colaborativas – será possível entender o fenômeno da dor neonatal^(6,7,8)^.

Objetiva-se, com base no exposto, produzir um ensaio teórico que reflita sobre a complexidade da dor à luz do pensamento sistêmico de Fritjof Capra. O referido autor concebe a complexidade como a mudança de uma visão cartesiana e reducionista para uma visão sistêmica, onde o mundo é visto como um todo integrado, em uma teia dinâmica de eventos interrelacionados.

## MÉTODO

Estudo teórico-reflexivo que analisa o fenômeno da dor em RNs internados em UTIN, sob a perspectiva sistêmica, com o objetivo de embasar a construção de cuidados que favoreçam o seu conforto e bem-estar singular e multidimensional. O pensamento sistêmico-complexo estabelece-se como um referencial relevante à compreensão da multidimensionalidade da percepção dolorosa pelos RNs internados em UTIN e pelas equipes assistenciais, visando ao cuidado integrado, multidisciplinar, humanizado e sensível às particularidades do RN e seu ambiente.

Fritjof Capra, físico e pensador conhecido por promover o pensamento sistêmico, enfatiza a importância de entender os seres vivos como complexos, contextuais e únicos, fazendo parte de uma teia de relações, onde há um entrelaçamento e interdependência de todos os fenômenos. Ele enfatiza que, para entendermos a dor e sermos capazes de aliviá-la, devemos considerá-la em seu contexto mais amplo, que inclui não só sua base física, mas também a emocional e ambiental, além de suas interconexões.

Assim, o presente estudo delineia-se com base no arcabouço teórico-reflexivo do pensamento sistêmico de produções de Fritjof Capra^(9,10)^ e autores de referência sistêmica citados em suas obras, a fim de fundamentar uma linha de cuidados neonatais mais humanizada, pluridimensional, singular e interativa, e que se faz através de uma ação multiprofissional integrada.

## A DOR NEONATAL: EXPERIÊNCIA COMPLEXA

O estudo da dor neonatal é relativamente recente. Somente após 1980 foi demonstrado que o RN é capaz de sentir dor, inclusive o prematuro extremo, rompendo com o paradigma de que por possuir órgão e conexões imaturas não seria capaz de vivenciar essa experiência. Desde então, muitos trabalhos têm sido publicados mostrando as consequências resultantes da exposição prolongada e não manejada a estímulos álgicos, além de formas de melhor identificá-la e manejá-la^(1,2,11)^.

No estudo da dor, a própria característica do sistema nervoso com sua intrincada rede de neurônios e vias de comunicação corroboram com a necessidade de uma visão sistêmica do tema. Suas múltiplas conexões e mecanismos de retroalimentação tornam a experiência dolorosa única para cada ser humano. Dada sua complexidade, a dor não pode ser restrita somente a um fenômeno celular físico, mas sim a uma rede de interações entre as esferas biológicas, emocionais, psicológicas e sociais^(2)^. Assim, seu estudo transcende o âmbito de uma estrutura reducionista, exigindo uma visão que considere a totalidade do indivíduo e suas interações com o ambiente^(12,13)^.

Os mecanismos envolvidos no fenômeno da dor, ou nociceptivo são considerados respostas adaptativas do corpo a algo potencialmente danoso. Essas respostas ocorrem após estímulo de neurônios nociceptivos sensíveis a estímulos térmicos, mecânicos ou químicos, que contém e liberam neuropeptídeos e sofrem influências hormonais e imunes. Para que um estímulo nociceptivo seja percebido, são necessárias quatro etapas: transdução (onde nociceptores periféricos transformam os estímulos álgicos em potenciais de ação), transmissão (através dos nervos periféricos para coluna vertebral), percepção (através do trato espinotalâmico, o estímulo chega até o córtex cerebral, onde é percebido como dor) e modulação (estímulo descendente que age modulando a intensidade do estímulo ascendente, agindo como opioides endógenos)^(14)^. Assim, o sistema nociceptivo possui a importante função de prover ao corpo a habilidade de responder a qualquer estímulo potencialmente danoso, sendo crucial para a sobrevivência e interação com o ambiente^(15)^.

Os estímulos dolorosos ou estressantes induzem a uma resposta fisiológica, que inclui o aumento de catecolaminas circulantes, da pressão arterial e intracraniana e da frequência cardíaca, o que demonstra a interconexão de todos os sistemas na percepção dolorosa. Em prematuros, essa resposta é pouco competente, o que torna os indícios de alterações comportamentais e de sinais vitais pouco confiáveis no reconhecimento da dor. Soma-se a isso o fato de que estímulos dolorosos persistentes exaurem a resposta do sistema nervoso simpático, obscurecendo os sinais de dor ou desconforto^(1,2,16)^.

O impacto da dor neonatal não manejada reafirma sua característica sistêmica, pois ocasiona desde alterações de sensibilidade local, baixas taxas de crescimento até alterações estruturais cerebrais, como: redução de substância branca, cerebelar e talâmica, com consequentes piores resultados cognitivos, motores e visuais. Soma-se a essas situações, piores desfechos clínicos, com consequente aumento de morbimortalidade. Com isso, torna-se imperativo uma abordagem sistêmica do assunto, como uma importante medida de redução da dor neonatal e da mortalidade infantil^(17,18,19)^.

São muitos os estímulos capazes de serem percebidos como dolorosos pelos neonatos, que abrangem desde procedimentos invasivos (punções, cateterismos, sondagens), patologias associadas (enterocolite, síndrome de aspiração de mecônio, pneumonias, infecções), até fatores ambientais (luz excessiva, ruídos), além do próprio ato de cuidado realizado pela equipe assistencial. Os prematuros extremos (nascidos com menos de 28 semanas de idade gestacional) são uma população de risco ainda maior para procedimentos dolorosos repetidos, em função do seu tempo de permanência na UTIN e de suas patologias associadas à prematuridade. Assim, faz-se necessário o uso de uma estratégia de melhoria de cuidados capaz de levar em consideração todos esses aspectos do estímulo doloroso^(1)^.

A natureza não verbal do RN levou ao desenvolvimento de escalas para auxiliar na identificação do processo doloroso, que consideram as alterações fisiológicas e comportamentais que podem advir do estímulo álgico. Elas são uma parte da avaliação, e não o todo, e devem ser aplicadas à luz do contexto em que se encontra o RN, pois os mais imaturos podem ter uma resposta abolida pela própria imaturidade ou até mesmo pela exposição crônica à dor. Além disso, deve-se lembrar que o grau de conhecimento da equipe assistencial também influencia nesse manejo^(2,4)^.

A Política Nacional de Atenção Humanizada ao Recém-Nascido – Método Canguru, estabelecida pelo Ministério da Saúde, preconiza um modelo de atenção perinatal voltado para a qualidade do cuidado, o acolhimento e a humanização da assistência. Esta política reconhece que o cuidado neonatal deve ir além dos aspectos biomédicos, incorporando dimensões emocionais, familiares e ambientais. No que se refere ao manejo da dor neonatal, a política enfatiza a necessidade de estratégias integradas que incluam medidas não farmacológicas, como o contato pele a pele, a amamentação, a sucção não nutritiva e a redução de estímulos nocivos, além das intervenções farmacológicas quando necessárias. O documento ressalta ainda a importância da capacitação permanente das equipes de saúde para a identificação precoce e o manejo adequado da dor, considerando as particularidades de cada recém-nascido e promovendo uma ambiência favorável ao desenvolvimento neuropsicomotor^(20)^. Essa abordagem está alinhada com os princípios da neuroproteção, que visam minimizar lesões cerebrais e otimizar o desenvolvimento do sistema nervoso central durante o período de internação^(21)^. Apesar disso, hoje as escalas da dor validadas não incorporam na sua métrica a ambiência de cada RN, o que pode levar a erros na identificação e manejo dos fenômenos álgicos.

Assim, faz-se necessário um instrumento multidimensional, construído na ótica do pensamento da complexidade, que seja flexível, permitindo adaptações em tempo real e considerando fatores fisiológicos, emocionais e ambientais, a ser usado como guia pela equipe assistencial. A implementação de estratégias de manejo deve ser multidisciplinar, promovendo uma abordagem colaborativa que reconheça a interdependência dos fatores envolvidos na experiência dolorosa^(6,7,8)^.

A adoção de uma perspectiva complexa na avaliação da dor neonatal demanda uma mudança de paradigma na prática clínica, com a capacitação de uma equipe capaz de entender e atuar sobre a rede de interações que compõem o sistema neonatal (Figura 1).

**Figura 1:** Características da dor neonatal.

Fonte: Elaborado pela autora.

## A DOR NEONATAL NA PERSPECTIVA DO PENSAMENTO SISTÊMICO

No pensar do físico e teórico dos sistemas, Fritjof Capra, só é possível entender os fenômenos vivos por meio de uma mudança de paradigma, desconstruindo a visão mecanicista, cartesiana e reducionista de Descartes e Newton e reconstruindo uma visão do mundo sistêmica. Nessa nova visão ou abordagem, o mundo é visto como um todo integrado e não como uma coleção de partes dissociadas, por meio da interdependência fundamental de todos os fenômenos^(9)^.

A visão sistêmica da vida, chamada novo paradigma, versa sobre uma visão holística do mundo, ou melhor, ecológica profunda, onde os sistemas vivos, além de serem vistos como um todo integrado e não somente uma coleção de partes, encontram-se encaixados no seu ambiente natural e social, física e espiritualmente, em um equilíbrio dinâmico. Há um entrelaçamento e interdependência de todos os fenômenos, por meio da concepção dos sistemas vivos como compostos por redes, onde não há hierarquias, somente redes aninhadas dentro de outras redes. O universo é visto como uma teia dinâmica de eventos interrelacionados, onde todas as concepções e teorias científicas são limitadas e aproximadas^(10)^.

Em se tratando do sistema nervoso humano, Capra o descreve como dinâmico e em contínua modificação, através da interação das informações processadas com o meio ambiente. Sendo assim, a inteligência, a memória e as decisões humanas nunca são completamente racionais, mas coloridas por emoções, com todos os saberes a partir da experiência, ou seja, únicas de cada indivíduo. Já a organização circular do sistema nervoso e sua característica auto-organizadora e autorreferente, leva ao entendimento de que as percepções não podem ser vistas como meras representações de uma realidade externa, mas entendidas como a criação contínua de novas relações dentro da rede neural^(22,23)^.

No que se refere à cognição, ou seja, o processo do conhecer, temos a atividade mental como a atividade organizadora dos sistemas vivos em todos os níveis. Ela se manifesta não apenas em organismos individuais, mas também em sistemas sociais e ecossistemas. Assim, a teoria de Santiago, desenvolvida por Maturana e Varela, que diz que não é necessário um cérebro para que a mente exista, ampliando a concepção do pensar e englobando todo o processo que envolve percepção, emoção e ação. Nessa premissa, até os organismos mais simples são capazes de cognição, reagindo ao meio em que estão^(9)^.

O papel do estresse e dos estados emocionais no curso das doenças já é hoje bem documentado, sendo a equipe assistencial responsável por lidar com o paciente como um todo e com sua relação com o meio ambiente físico e emocional. O próprio fenômeno da dor ainda é pouco entendido devido às limitações dos cientistas e profissionais de saúde em integrar elementos físicos e psicológicos. As pesquisas ainda apresentam lacunas relacionadas aos fatores precisos da causa da dor, razão pela qual não são ainda bem compreendidas as vias de comunicação entre o corpo e a mente. Na prática, é quase sempre impossível distinguir quais as fontes de dor biológica e quais as fontes de dor psicológica de dois pacientes com os mesmos sintomas físicos, podendo um estar sofrendo dores excruciantes enquanto outro pouco sente^(24)^.

Assim, para melhor entender e, consequentemente, melhor manejar o fenômeno da dor, deve-se considerá-la em seu contexto mais amplo, que inclui atitudes, expectativas mentais do paciente, seu sistema de crenças, apoio da família e muitas outras circunstâncias. A atual prática médica tende a limitar a dor a algum distúrbio fisiológico específico, onde muitas vezes é negada ou suprimida com analgésicos somente. Logo, a redução do estresse, além dos processos álgicos, deve compor o tratamento, visando o processo da cura^(9)^.

A permanência de uma visão patriarcal de poder no sistema de assistência à saúde, centrada na figura do médico, impede frequentemente que o restante da equipe use todo seu potencial para o processo da cura. O importante papel que as enfermeiras desempenham, por exemplo, no processo de cura, por meio do contato próximo com os pacientes, não é plenamente reconhecido. Daí a importância de uma abordagem multidisciplinar e abrangente, que vá além da avaliação científica, mas que considere o conhecimento mais amplo do estado físico e psicológico, através de uma visão integrada do estado emocional, histórico familiar e situação social do paciente^(6,7,8,9,25)^.

Na ciência sistêmica, os sistemas vivos não podem ser compreendidos por meio da análise, pois as propriedades das partes não são propriedades intrínsecas, e só podem ser compreendidas dentro do contexto do todo maior. Dessa forma, o pensamento sistêmico é contextual e ambientalista, ao levar em consideração o ambiente em que determinado sistema está inserido. Nele, ocorrem múltiplas interconexões, *feedbacks* e processos não lineares, que produzem comportamentos emergentes impossíveis de serem previstos por uma análise fragmentada^(10)^. Seguindo essa abordagem, a dor deve ser estudada e avaliada como um fenômeno integrado, que sofre influências do meio externo, necessitando para sua melhor compreensão uma abordagem que considere a sua complexidade, onde fatores fisiológicos, neurológicos, ambientais e emocionais estejam interligados (Quadro 1).

**Quadro 1:** Paralelo entre as visões reducionistas e da complexidade – Santa Maria, RS, Brasil, 2025.

Aspecto	Visão reducionista	Visão da complexidade
**Perspectiva geral**	Enxerga a dor como um fenômeno simples, linear e isolado.	Enxerga a dor como um fenômeno complexo, multifacetado e interconectado.
**Base teórica**	Influenciada pela física clássica de Newton, que vê o mundo como causa e efeito direto.	Inspirada na teoria sistêmica, que valoriza as interações e a interdependência dos elementos.
**Abordagem ao tratamento**	Foca em intervenções pontuais, mecânicas e específicas.	Adota uma abordagem multidisciplinar, considerando fatores fisiológicos, emocionais, sociais e ambientais.
**Reconhecimento da dor**	Subestima ou negligencia a experiência subjetiva do RN.	Reconhece a dor como uma experiência complexa, influenciada por diversos fatores contextuais.
**Cuidado ao RN**	Trata o RN como um sistema mecânico, sem considerar seu ambiente ou emoções.	Enxerga o RN como um ser integral, com necessidades emocionais, sociais e ambientais.
**Prática clínica**	Intervenções isoladas, muitas vezes sem considerar o contexto completo.	Intervenções integradas, humanizadas e sensíveis às particularidades do RN e seu ambiente.
**Exemplo de estratégia**	Uso de medicamentos específicos para aliviar a dor.	Uso de estratégias multidisciplinares, incluindo cuidados ambientais, emocionais e fisiológicos.

Fonte: Elaborado pela autora.

Aplicando esses conceitos no ecossistema neonatal, pode-se entender o porquê de a dor neonatal não poder ser reduzida às alterações isoladas de sinais fisiológicos ou a uma única causa. Ela deve ser percebida como produto de uma intrincada rede de interações entre o sistema nervoso do RN, o ambiente hospitalar em que está inserido, as ações da equipe assistencial e o contexto emocional do próprio RN e da família. Logo, estímulos sensoriais excessivos ou inadequados, como luzes fortes e ruídos, podem levar à alteração do estado fisiológico do RN, que por sua vez podem influenciar sua resposta comportamental e sua experiência subjetiva da dor. Além disso, o *feedback* entre esses elementos também pode criar um ciclo dinâmico onde mudanças pequenas podem gerar efeitos amplificados ou atenuados ao longo do tempo. Compreender o sistema neonatal como um todo promove ações que favorecem a estabilidade, o conforto e o bem-estar do RN, levando em conta suas interconexões.

## CONCLUSÃO

A dor devido às suas características complexas, pode não estar sendo identificada eficazmente através da simples aplicação de escalas, sem levar em consideração os demais componentes do ecossistema neonatal, que acabam não sendo contemplados nas métricas validadas atualmente e utilizadas nas UTINs.

A enfermagem neonatal ocupa uma posição estratégica na identificação, avaliação e manejo da dor, devido à sua proximidade contínua com o recém-nascido e sua família. Para isso, deve-se desenvolver competências para uma avaliação contextualizada da dor, que transcenda a aplicação mecânica de escalas e incorpore a observação criteriosa do ambiente, das respostas comportamentais sutis e da trajetória clínica, o que exige formação continuada e competência clínica para reconhecer sinais atípicos, especialmente em prematuros extremos e neonatos gravemente enfermos. Ao liderar também a implementação de estratégias não farmacológicas e a articulação e coordenação de ações multidisciplinares, assume-se o protagonismo na redução do sofrimento neonatal e na promoção de melhores desfechos a curto e longo prazo.

A adoção do pensamento sistêmico na compreensão da dor neonatal implica repensar estratégias de avaliação e manejo, incentiva a formação de equipes de cuidados que atuem de forma colaborativa e que reconheçam a importância de cada elemento do sistema na experiência da dor neonatal, além de promover o desenvolvimento de pesquisas que levem em conta a característica complexa desse fenômeno, integrando aspectos físicos, sociais e emocionais na percepção álgica. Capra, nesse sentido, fornece uma base teórica sólida para esta compreensão, ao enfatizar a interconexão, a dinâmica e a emergência dos sistemas, considerando o RN como um sistema completo, em constante interação com seu ambiente.

## DISPONIBILIDADE DE DADOS

Todo o conjunto de dados que dá suporte aos resultados deste estudo foi publicado no próprio artigo.

# Translation: El dolor neonatal bajo una perspectiva amplificada: análisis a la luz del pensamiento sistémico

**DOI:** 10.1590/1980-220X-REEUSP-2025-0399es

**Authors:** Silvia Naujorks, Julieli Rosso, Luana Pizarro Meneghello, Dirce Stein Backs, Regina Gema Santini Costenaro

**Affiliations:** 1 Universidade Franciscana, Santa Maria, RS, Brasil.

**Keywords:** Dolor, Recién Nacido, Unidades de Cuidado Intensivo Neonatal, Promoción de la Salud.

## Abstract

El objetivo es reflexionar sobre la complejidad del dolor neonatal a la luz del pensamiento sistémico de Fritjof Capra. Es un ensayo teórico que analiza el fenómeno del dolor en recién nacidos ingresados en terapia intensiva neonatal, su carácter complejo y multidimensional. Se observan las principales características del sistema neonatal que conducen y sustentan la atención multiprofesional desde una perspectiva sistémica. Se concibe un paralelismo entre la visión reduccionista, esbozada por intervenciones puntuales y aisladas, y la visión del dolor desde la perspectiva de la complejidad, que conduce a la integralidad, la multidisciplinariedad y la interconectividad. El pensamiento sistémico-complejo se establece como un referente para comprender la percepción del dolor de los recién nacidos y del personal sanitario, buscando una atención integrada, multidisciplinar, humanizada y sensible a sus particularidades y al entorno. Las implicaciones de la enfermería neonatal incluyen la necesidad de una evaluación continua y contextualizada del dolor, el desarrollo de competencias para la conducción integrada y el liderazgo en la coordinación de estrategias multidisciplinarias.

## INTRODUCCIÓN

La Asociación Internacional del Estudio del Dolor (IASP) definió en 2020 el dolor como una experiencia sensorial y emocional desagradable, no necesariamente asociada a una lesión tisular real. Se trata de una experiencia compleja, resultado de la interacción de los sistemas inmunológico, nervioso y endocrino, que puede verse influida por experiencias dolorosas previas^(1)^.

Los recién nacidos (RN) ingresados en la Unidad de Terapia Intensiva Neonatal (UTIN) son una población de riesgo debido a que se someten diariamente a procedimientos potencialmente dolorosos durante su hospitalización. Además, su naturaleza no verbal y su inmadurez dificultan la correcta identificación y manejo de estos estímulos algicos, lo que da lugar a altos índices de procedimientos potencialmente dolorosos realizados sin ningún tipo de analgesia^(2)^.

Son muchos los estudios que demuestran las consecuencias a corto y largo plazo que pueden tener los estímulos dolorosos repetidos y no tratados, desde bajas tasas de crecimiento hasta alteraciones en el desarrollo cerebral, cognitivo, motor y visual, lo que conduce a peores resultados en la edad escolar^(3,4)^. A esto se suman los efectos inmediatos de los estímulos dolorosos, que incluyen taquicardia, aumento de la presión arterial, alteración de los factores de coagulación y estrés oxidativo que, en conjunto, pueden aumentar el riesgo de hemorragia intracraneal y mortalidad^(1)^.

Así, dada la vulnerabilidad de esta población, corresponde al equipo de profesionales de la salud que atiende al recién nacido (RN) tener una visión ampliada y colegiada, a fin de comprender la singularidad y la multidimensionalidad terapéutica del dolor, a través de su calificación y fundamentación en referencias sistémicas^(5,6)^.

El pensamiento sistémico considera enfoques interprofesionales, a partir de una comprensión singular y multidimensional. Solo con un enfoque amplio, que considere la interdependencia y el entrelazamiento de todos los fenómenos en el sistema neonatal, que sea multidisciplinario y con estrategias de manejo colaborativas, será posible comprender el fenómeno del dolor neonatal^(6,7,8)^.

Con base en lo expuesto, el objetivo es producir un ensayo teórico que reflexione sobre la complejidad del dolor a la luz del pensamiento sistémico de Fritjof Capra. Dicho autor concibe la complejidad como el cambio de una visión cartesiana y reduccionista a una visión sistémica, donde el mundo se ve como un todo integrado, en una red dinámica de eventos interrelacionados.

## MÉTODO

Estudio teórico-reflexivo que analiza el fenómeno del dolor en los recién nacidos ingresados en la UTIN, desde una perspectiva sistémica, con el objetivo de sentar las bases para la construcción de cuidados que favorezcan su comodidad y bienestar singular y multidimensional.

El pensamiento sistémico-complejo se establece como un referente relevante para comprender la multidimensionalidad de la percepción del dolor por parte de los recién nacidos ingresados en la UTIN y los equipos de asistencia, con el objetivo de proporcionar una atención integrada, multidisciplinaria, humanizada y sensible a las particularidades del recién nacido y su entorno.

Fritjof Capra, físico y pensador conocido por promover el pensamiento sistémico, enfatiza la importancia de entender a los seres vivos como complejos, contextuales y únicos, formando parte de una red de relaciones, donde existe un entrelazamiento e interdependencia de todos los fenómenos. Destaca que, para comprender el dolor y ser capaces de aliviarlo, debemos considerarlo en su contexto más amplio, que incluye no solo su base física, sino también la emocional y ambiental, además de sus interconexiones.

Así, el presente estudio se basa en el marco teórico-reflexivo del pensamiento sistémico de las producciones de Fritjof Capra^(9,10)^ y autores de referencia sistémica citados en sus obras, con el fin de fundamentar una línea de cuidados neonatales más humanizada, multidimensional, singular e interactiva, que se lleva a cabo a través de una acción multiprofesional integrada.

## EL DOLOR NEONATAL: UNA EXPERIENCIA COMPLEJA

El estudio del dolor neonatal es relativamente reciente. Solo después de 1980 se demostró que el recién nacido es capaz de sentir dolor, incluso el prematuro extremo, rompiendo con el paradigma de que, al tener órganos y conexiones inmaduras, no sería capaz de experimentar esta sensación. Desde entonces, se han publicado muchos trabajos que muestran las consecuencias de la exposición prolongada y no controlada a estímulos dolorosos, así como formas de identificarlo y manejarlo mejor^(1,2,11)^.

En el estudio del dolor, la propia característica del sistema nervioso, con su intrincada red de neuronas y vías de comunicación, corrobora la necesidad de una visión sistémica del tema. Sus múltiples conexiones y mecanismos de retroalimentación hacen que la experiencia dolorosa sea única para cada ser humano. Dada su complejidad, el dolor no puede restringirse únicamente a un fenómeno celular físico, sino que es una red de interacciones entre las esferas biológica, emocional, psicológica y social^(2)^. Por lo tanto, su estudio trasciende el ámbito de una estructura reduccionista y exige una visión que considere la totalidad del individuo y sus interacciones con el entorno^(12,13)^.

Los mecanismos involucrados en el fenómeno del dolor, o nociceptivo, se consideran respuestas adaptativas del cuerpo a algo potencialmente dañino. Estas respuestas se producen tras el estímulo de neuronas nociceptivas sensibles a estímulos térmicos, mecánicos o químicos, que contienen y liberan neuropéptidos y sufren influencias hormonales e inmunitarias. Para que se perciba un estímulo nociceptivo, son necesarias cuatro etapas: transducción (donde los nociceptores periféricos transforman los estímulos algicos en potenciales de acción), transmisión (a través de los nervios periféricos a la columna vertebral), percepción (a través del tracto espinotalámico, el estímulo llega al córtex cerebral, donde se percibe como dolor) y modulación (estímulo descendente que actúa modulando la intensidad del estímulo ascendente, actuando como opioides endógenos)^(14)^. Así, el sistema nociceptivo tiene la importante función de proporcionar al cuerpo la capacidad de responder a cualquier estímulo potencialmente dañino, lo que es crucial para la supervivencia y la interacción con el entorno^(15)^.

Los estímulos dolorosos o estresantes inducen una respuesta fisiológica, que incluye el aumento de las catecolaminas circulantes, la presión arterial e intracraneal y la frecuencia cardíaca, lo que demuestra la interconexión de todos los sistemas en la percepción del dolor.

En los prematuros, esta respuesta es poco competente, lo que hace que los indicios de alteraciones conductuales y de signos vitales sean poco fiables para reconocer el dolor. A esto se suma el hecho de que los estímulos dolorosos persistentes agotan la respuesta del sistema nervioso simpático, oscureciendo los signos de dolor o malestar^(1,2,16)^.

El impacto del dolor neonatal no tratado reafirma su carácter sistémico, ya que provoca desde alteraciones de la sensibilidad local y bajas tasas de crecimiento hasta alteraciones estructurales cerebrales, como la reducción de la sustancia blanca, cerebelosa y talámica, con el consiguiente empeoramiento de los resultados cognitivos, motores y visuales. A estas situaciones se suman peores resultados clínicos, con el consiguiente aumento de la morbimortalidad. Por ello, es imperativo abordar el tema de manera sistémica, como una medida importante para reducir el dolor neonatal y la mortalidad infantil^(17,18,19)^.

Son muchos los estímulos que pueden ser percibidos como dolorosos por los recién nacidos, que abarcan desde procedimientos invasivos (punciones, cateterismos, sondajes), patologías asociadas (enterocolitis, síndrome de aspiración de meconio, neumonías, infecciones), hasta factores ambientales (luz excesiva, ruidos), además del propio acto de cuidado realizado por el equipo asistencial. Los prematuros extremos (nacidos con menos de 28 semanas de edad gestacional) son una población de riesgo aún mayor para procedimientos dolorosos repetidos, debido a su tiempo de permanencia en la UTIN y a sus patologías asociadas a la prematuridad. Por lo tanto, es necesario utilizar una estrategia de mejora de los cuidados que tenga en cuenta todos estos aspectos del estímulo doloroso^(1)^.

La naturaleza no verbal del recién nacido ha llevado al desarrollo de escalas para ayudar a identificar el proceso doloroso, que tienen en cuenta los cambios fisiológicos y conductuales que pueden derivarse del estímulo doloroso. Estas escalas son una parte de la evaluación, no el todo, y deben aplicarse teniendo en cuenta el contexto en el que se encuentra el recién nacido, ya que los más inmaduros pueden tener una respuesta abolida por su propia inmadurez o incluso por la exposición crónica al dolor. Además, hay que recordar que el grado de conocimiento del equipo asistencial también influye en este manejo^(2,4)^.

La Política Nacional de Atención Humanizada al Recién Nacido – Método Canguro, establecida por el Ministerio de Salud, recomienda un modelo de atención perinatal orientado a la calidad del cuidado, la acogida y la humanización de la asistencia. Esta política reconoce que la atención neonatal debe ir más allá de los aspectos biomédicos, incorporando dimensiones emocionales, familiares y ambientales. En lo que respecta al manejo del dolor neonatal, la política enfatiza la necesidad de estrategias integradas que incluyan medidas no farmacológicas, como el contacto piel con piel, la lactancia materna, la succión no nutritiva y la reducción de estímulos nocivos, además de las intervenciones farmacológicas cuando sean necesarias. El documento también destaca la importancia de la capacitación permanente de los equipos de salud para la identificación temprana y el manejo adecuado del dolor, considerando las particularidades de cada recién nacido y promoviendo un ambiente favorable para el desarrollo neuropsicomotor^(20)^. Este enfoque está en consonancia con los principios de la neuroprotección, que tienen por objeto minimizar las lesiones cerebrales y optimizar el desarrollo del sistema nervioso central durante el período de hospitalización^(21)^. A pesar de ello, en la actualidad las escalas de dolor validadas no incorporan en su métrica el entorno de cada recién nacido, lo que puede dar lugar a errores en la identificación y el manejo de los fenómenos algicos.

Por lo tanto, es necesario un instrumento multidimensional, construido desde la perspectiva del pensamiento de la complejidad, que sea flexible, permita adaptaciones en tiempo real y tenga en cuenta factores fisiológicos, emocionales y ambientales, para que lo utilice como guía el equipo asistencial. La implementación de estrategias de manejo debe ser multidisciplinaria, promoviendo un enfoque colaborativo que reconozca la interdependencia de los factores involucrados en la experiencia dolorosa^(6,7,8)^.

La adopción de una perspectiva compleja en la evaluación del dolor neonatal exige un cambio de paradigma en la práctica clínica, con la capacitación de un equipo capaz de comprender y actuar sobre la red de interacciones que componen el sistema neonatal (Figura 1).

**Figura 1:** Características del dolor neonatal.

Fuente: Elaborado por la autora.

## EL DOLOR NEONATAL DESDE LA PERSPECTIVA DEL PENSAMIENTO SISTÉMICO

Según el físico y teórico de sistemas Fritjof Capra, solo es posible comprender los fenómenos vivos mediante un cambio de paradigma, deconstruyendo la visión mecanicista, cartesiana y reduccionista de Descartes y Newton y reconstruyendo una visión sistémica del mundo. En esta nueva visión o enfoque, el mundo se ve como un todo integrado y no como una colección de partes disociadas, a través de la interdependencia fundamental de todos los fenómenos^(9)^.

La visión sistémica de la vida, llamada nuevo paradigma, trata sobre una visión holística del mundo, o mejor dicho, profundamente ecológica, en la que los sistemas vivos, además de ser vistos como un todo integrado y no solo como una colección de partes, se encuentran integrados en su entorno natural y social, física y espiritualmente, en un equilibrio dinámico. Existe un entrelazamiento y una interdependencia de todos los fenómenos, a través de la concepción de los sistemas vivos como compuestos por redes, donde no hay jerarquías, solo redes anidadas dentro de otras redes. El universo se ve como una red dinámica de eventos interrelacionados, donde todas las concepciones y teorías científicas son limitadas y aproximadas^(10)^.

En lo que respecta al sistema nervioso humano, Capra lo describe como dinámico y en continua modificación, a través de la interacción de la información procesada con el medio ambiente. Por lo tanto, la inteligencia, la memoria y las decisiones humanas nunca son completamente racionales, sino que están teñidas de emociones, con todos los conocimientos basados en la experiencia, es decir, únicos para cada individuo. Por otra parte, la organización circular del sistema nervioso y su característica autoorganizadora y autorreferencial lleva a entender que las percepciones no pueden verse como meras representaciones de una realidad externa, sino como la creación continua de nuevas relaciones dentro de la red neural^(22,23)^.

En lo que respecta a la cognición, es decir, el proceso de conocer, tenemos la actividad mental como la actividad organizadora de los sistemas vivos en todos los niveles. Se manifiesta no solo en organismos individuales, sino también en sistemas sociales y ecosistemas. Así, la teoría de Santiago, desarrollada por Maturana y Varela, afirma que no es necesario un cerebro para que exista la mente, ampliando la concepción del pensamiento y abarcando todo el proceso que implica la percepción, la emoción y la acción. Según esta premisa, incluso los organismos más simples son capaces de cognición, reaccionando al medio en el que se encuentran.

El papel del estrés y los estados emocionales en el curso de las enfermedades ya está bien documentado, siendo el equipo asistencial responsable de tratar al paciente en su totalidad y su relación con el entorno físico y emocional. El propio fenómeno del dolor aún se comprende poco debido a las limitaciones de los científicos y profesionales de la salud para integrar elementos físicos y psicológicos. Las investigaciones aún presentan lagunas relacionadas con los factores precisos que causan el dolor, por lo que aún no se comprenden bien las vías de comunicación entre el cuerpo y la mente. En la práctica, casi siempre es imposible distinguir cuáles son las fuentes de dolor biológico y cuáles son las fuentes de dolor psicológico de dos pacientes con los mismos síntomas físicos, ya que uno puede estar sufriendo dolores insoportables mientras que el otro apenas siente nada^(24)^.

Por lo tanto, para comprender mejor y, en consecuencia, manejar mejor el fenómeno del dolor, se debe considerar en su contexto más amplio, que incluye las actitudes, las expectativas mentales del paciente, su sistema de creencias, el apoyo de la familia y muchas otras circunstancias. La práctica médica actual tiende a limitar el dolor a algún trastorno fisiológico específico, donde a menudo se niega o se suprime solo con analgésicos. Por lo tanto, la reducción del estrés, además de los procesos algicos, debe formar parte del tratamiento, con el objetivo de lograr la curación^(9)^.

La persistencia de una visión patriarcal del poder en el sistema de asistencia sanitaria, centrada en la figura del médico, impide a menudo que el resto del equipo utilice todo su potencial para el proceso de curación. El importante papel que desempeñan las enfermeras, por ejemplo, en el proceso de curación, a través del contacto cercano con los pacientes, no se reconoce plenamente. De ahí la importancia de un enfoque multidisciplinario y amplio, que vaya más allá de la evaluación científica, pero que considere un conocimiento más amplio del estado físico y psicológico, a través de una visión integrada del estado emocional, los antecedentes familiares y la situación social del paciente^(6,7,8,9,25)^.

En la ciencia sistémica, los sistemas vivos no pueden entenderse mediante el análisis, ya que las propiedades de las partes no son propiedades intrínsecas y solo pueden entenderse dentro del contexto del todo mayor. De este modo, el pensamiento sistémico es contextual y ambientalista, al tener en cuenta el entorno en el que se inserta un determinado sistema. En él se producen múltiples interconexiones, feedbacks y procesos no lineales, que producen comportamientos emergentes imposibles de predecir mediante un análisis fragmentado^(10)^. Siguiendo este enfoque, el dolor debe estudiarse y evaluarse como un fenómeno integrado, que sufre influencias del medio externo y que, para su mejor comprensión, requiere un enfoque que tenga en cuenta su complejidad, en el que los factores fisiológicos, neurológicos, ambientales y emocionales estén interrelacionados (Cuadro 1).

**Cuadro 1:** Comparación entre las visiones reduccionistas y las de la complejidad – Santa Maria, RS, Brasil, 2025.

Aspecto	Visión reduccionista	Visión de la complejidad
**Perspectiva general**	Mira el dolor como un fenómeno simples, linear y aislado.	Mira el dolor como un fenómeno complexo, multifacético e interconectado.
**Base teórica**	Influenciada pela física clásica de Newton, que ve el mundo como causa y efecto directo.	Inspirada en la teoría sistémica, que valoriza las interacciones y la interdependencia de los elementos.
**Abordaje para el tratamiento**	Enfoca en intervenciones puntuales, mecánicas y específicas.	Adopta un abordaje multidisciplinario, considerando factores fisiológicos, emocionales, sociales y ambientales.
**Reconocimiento del dolor**	Subestima o negligencia la experiencia subjetiva del RN.	Reconoce el dolor como una experiencia compleja, influenciada por diversos factores contextuales.
**Cuidado al RN**	Trata al RN como un sistema mecánico, sin considerar su ambiente o emociones.	Mira al RN como un ser integral, con necesidades emocionales, sociales y ambientales.
**Práctica clínica**	Intervenciones aisladas, muchas veces sin considerar el contexto completo.	Intervenciones integradas, humanizadas y sensibles a las particularidades del RN y su ambiente.
**Ejemplo de estrategia**	Uso de medicamentos específicos para aliviar el dolor.	Uso de estrategias multidisciplinares, incluyendo cuidados ambientales, emocionales y fisiológicos.

Fuente: elaborado por la autora.

Al aplicar estos conceptos al ecosistema neonatal, se puede entender por qué el dolor neonatal no puede reducirse a alteraciones aisladas de señales fisiológicas o a una única causa. Debe percibirse como el resultado de una intrincada red de interacciones entre el sistema nervioso del recién nacido, el entorno hospitalario en el que se encuentra, las acciones del equipo de atención y el contexto emocional del propio recién nacido y de la familia. Por lo tanto, los estímulos sensoriales excesivos o inadecuados, como las luces fuertes y los ruidos, pueden alterar el estado fisiológico del recién nacido, lo que a su vez puede influir en su respuesta conductual y en su experiencia subjetiva del dolor. Además, el feedback entre estos elementos también puede crear un ciclo dinámico en el que pequeños cambios pueden generar efectos amplificados o atenuados a lo largo del tiempo. Comprender el sistema neonatal en su conjunto promueve acciones que favorecen la estabilidad, la comodidad y el bienestar del recién nacido, teniendo en cuenta sus interconexiones.

## CONCLUSIÓN

Debido a sus características complejas, es posible que el dolor no se identifique de manera eficaz mediante la simple aplicación de escalas, sin tener en cuenta los demás componentes del ecosistema neonatal, que acaban no siendo contemplados en las métricas validadas actualmente y utilizadas en las UTIN.

La enfermería neonatal ocupa una posición estratégica en la identificación, evaluación y manejo del dolor, debido a su proximidad continua con el recién nacido y su familia. Para ello, se deben desarrollar competencias para una evaluación contextualizada del dolor, que trascienda la aplicación mecánica de escalas e incorpore la observación cuidadosa del entorno, las respuestas conductuales sutiles y la trayectoria clínica, lo que requiere formación continua y competencia clínica para reconocer signos atípicos, especialmente en prematuros extremos y neonatos gravemente enfermos. Al liderar también la implementación de estrategias no farmacológicas y la articulación y coordinación de acciones multidisciplinarias, se asume el protagonismo en la reducción del sufrimiento neonatal y en la promoción de mejores resultados a corto y largo plazo.

La adopción del pensamiento sistémico en la comprensión del dolor neonatal implica repensar las estrategias de evaluación y manejo, fomenta la formación de equipos de atención que actúen de manera colaborativa y que reconozcan la importancia de cada elemento del sistema en la experiencia del dolor neonatal, además de promover el desarrollo de investigaciones que tengan en cuenta la característica compleja de este fenómeno, integrando aspectos físicos, sociales y emocionales en la percepción del dolor. Capra, en este sentido, proporciona una base teórica sólida para esta comprensión, al enfatizar la interconexión, la dinámica y la emergencia de los sistemas, considerando al recién nacido como un sistema completo, en constante interacción con su entorno.

## DISPONIBILIDAD DE DATOS

Todo el conjunto de datos que apova los resultados de este estudio fue publicado en el propio artículo.
